# Gauge Factor and Stretchability of Silicon-on-Polymer Strain Gauges

**DOI:** 10.3390/s130708577

**Published:** 2013-07-05

**Authors:** Shixuan Yang, Nanshu Lu

**Affiliations:** Center for Mechanics of Solids, Structures and Materials, Department of Aerospace Engineering and Engineering Mechanics, University of Texas at Austin, Austin, TX 78712, USA; E-Mail: rock002008@utexas.edu

**Keywords:** piezoresistive, silicon, polymer substrate, gauge factor, stretchability

## Abstract

Strain gauges are widely applied to measure mechanical deformation of structures and specimens. While metallic foil gauges usually have a gauge factor slightly over 2, single crystalline silicon demonstrates intrinsic gauge factors as high as 200. Although silicon is an intrinsically stiff and brittle material, flexible and even stretchable strain gauges have been achieved by integrating thin silicon strips on soft and deformable polymer substrates. To achieve a fundamental understanding of the large variance in gauge factor and stretchability of reported flexible/stretchable silicon-on-polymer strain gauges, finite element and analytically models are established to reveal the effects of the length of the silicon strip, and the thickness and modulus of the polymer substrate. Analytical results for two limiting cases, *i.e.*, infinitely thick substrate and infinitely long strip, have found good agreement with FEM results. We have discovered that strains in silicon resistor can vary by orders of magnitude with different substrate materials whereas strip length or substrate thickness only affects the strain level mildly. While the average strain in silicon reflects the gauge factor, the maximum strain in silicon governs the stretchability of the system. The tradeoff between gauge factor and stretchability of silicon-on-polymer strain gauges has been proposed and discussed.

## 1. Introduction

Strain gauges are widely used across all engineering fields to measure mechanical deformation of a solid object. The most common type of strain gauges consists of a patterned metal foil on a stiff plastic backing sheet glued to the solid object. Deformation in the object leads to deformation in the foil, thereby causing its electrical resistance to change. The fractional change in resistance, Δ*R*/*R*_0_, is related to the mechanical strain *ε* by the gauge factor (*GF*):

(1) $$GF=\frac{\Delta R/R_{0}}{\varepsilon }$$

The *GF* for metallic foils are typically between 2 to 5 [1], due mostly to changes in length and cross-sectional area instead of changes in resistivity of the metal wires [2]. For precision measurements, however, semiconductor gauges are preferred over metal foils. In 1954 Smith first discovered the so-called piezoresistive effects [3], whereby the static resistance of a piece of semiconductor can be changed by a mechanical stress. In fact, it is the resistivity of the semiconductor that varies significantly with deformation, attributing to the strong dependence of the bandgap on inter-atomic spacing [3–5]. For example, the gauge factor of p-type (110) single crystalline silicon can be as high as 200 [3,6,7]. Since then, silicon-based microelectromechanical systems (MEMS) have found widespread use in load cells and pressure sensors [5]. Large piezoresistive coefficients, linear and instantaneous responses, multiplexing capabilities, and mature processing technologies represent attractive aspects of silicon for these applications. Due to the intrinsic stiffness and brittleness, however, semiconductor gauges are widely used as ‘hard’ sensors for small strain measurement on flat surfaces of stiff objects. If the surface of the target object is curvilinear, the gauge will be too stiff to completely conform to the surface. If the target object is too soft, the stiff gauge will significantly constrain the local deformation in the underlying region, resulting in understated strain measurement. If the target object is deforming too much, silicon will easily rupture.

Strain measurement on curvilinear surfaces and/or soft, highly deformable objects calls for flexible or even stretchable strain gauges. Examples of applications include structural health monitoring on curvilinear surfaces [8], multiplexed arrays of strain gauges for three-dimensional shape-mapping [9], gauges integrated on contact lenses for intraocular pressure monitoring [10], gauges mounted on human skin to monitor gait/joint motion [11–13], and gauges wrapped around beating heart to detect ischemia-induced heart stiffening [14]. Candidates of strain sensing materials for flexible/stretchable strain gauges include metals, silicon, piezoresistive elastomers, and even carbon nanotubes and graphene. Although carbon-doped elastomer exhibit intrinsic stretchability and strong piezoresistivity, the resistance of the percolated conductive networks is susceptible to drifting and hysteresis due to the viscoelasticity of rubber materials [15]. Inorganic materials generally offer better stability and repeatability, but their compliance and deformability are very limited. To make a gauge flexible or even stretchable, polymers are used as substrates to support thin wires or strips of strain sensing component. Metal wires exhibit much lower intrinsic gauge factors than silicon. Although flexible strain gauges based on carbon nanotubes [16] or graphene [17] have been reported, none has the maturity and proven effectiveness of silicon. As a result, we will focus on the mechanical responses of flexible and stretchable gauges based on polymer-supported thin silicon strips in this work.

Recent work shows that ultrathin sheets of single crystalline silicon, *i.e.*, silicon nanomembranes, can survive severe bending or stretch when supported by polymer substrates [18]. Depending on the substrate material, flexible strain gauges based on polyimide substrates [9] and tissue-like strain gauges based on elastomer substrates [13,14] have been reported. We notice that even silicon strips with the same thickness, orientation, and doping concentration, when bonded to different types of polymer substrates, the *GF* and stretchability (the applied strain beyond which silicon ruptures) can vary by orders of magnitude. For example, when polyimide substrates are used, *GF* obtained from uniaxial tension tests are 43 and the system cannot be stretched beyond 1% [9]. In contrast, when the substrate is elastomer, the measured *GF* reduced to 0.23 but the system can be stretched beyond 25% without inducing any cracks in silicon [13,14]. Mechanics models accounting for the silicon length and thickness as well as substrate modulus and thickness need to be developed to explain the discrepancies found in different systems and to guide the rationalized design of future flexible/stretchable silicon-on-polymer strain gauges.

In this paper, we describe finite element and analytical modeling of thin silicon strips bonded to polymer substrates of wide ranges of Young's modulus and thickness. Both gauge factor and stretchability can be predicted and effects of material and geometric variables are revealed. The tradeoff between *GF* and stretchability is proposed for the first time to provide guidance for the choice of substrate material and thickness. This paper is organized as follows: Section 2 describes the basic models adopted in this paper. Section 3 presents the finite element modeling (FEM) results. Section 4 provides analytical results for two limiting cases. Section 5 discusses the tradeoff between *GF* and stretchability. Concluding remarks are provided in Section 6.

## 2. Model Setup

**Figure 1.**
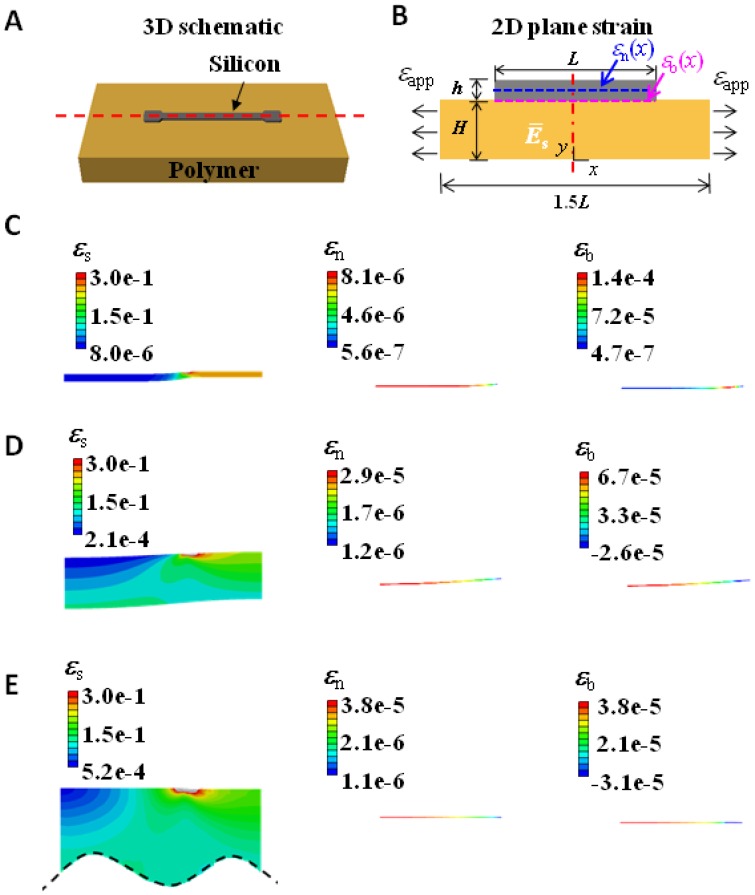
Schematic and FEM contour plots of a thin silicon strip supported by polymer substrate subject to uniaxial tension. (**A**) 3D schematic of a unit cell. (**B**) 2D plane strain model adopted in FEM. *ε*_n_(*x*) represents strain along the neutral axis of silicon and *ε*_b_(*x*) represents strain along the bottom surface of silicon. Due to symmetry, only the right half in (B) is modeled in FEM. (**C**) Contour plots of *ε*_s_ in substrate and *ε*_n_ and *ε*_b_ in silicon when *E*_s_ = 60 kPa and *H* = 30 μm, (**D**) *H* = 300 μm, and (**E**) *H* = 3,000 μm.

Silicon-based stretchable strain gauges are often composed of arrays of strips. The modeling work in this paper will just focus on a unit cell cut out of the periodic array. A schematic of a unit cell and its corresponding 2D plane strain model are depicted in Figure 1A,B. *L* represents the length of the silicon strip, *h* and *H* are the thicknesses of silicon and polymer, respectively. To minimize the number of variables, the size of the unit cell is fixed to be 1.5 *L* for all the models following a convention of island-on-polymer analysis [19–21]. When a uniform tensile strain, *ε*_app_, is applied to the substrate, the resistance of the silicon strip will change by Δ*R*, and the effective *GF* of the strain gauge system is defined as:

(2) $$GF=\frac{\Delta R/R_{0}}{\varepsilon_{\text{app}}}$$

Although strain distribution in silicon might not be uniform, it has been proven [11] that the fractional change of resistance is proportional to the average longitudinal strain in silicon, *i.e.*:

(3) $$\Delta R/R_{0}={\text{GF}}_{\text{Si}}\varepsilon_{\text{avg}}$$

where *GF*_Si_ represents the intrinsic gauge factor of silicon. Depending on the crystal orientation as well as the doping type and concentration [22], the *GF*_Si_ of p-type (110) silicon can reach as high as 200 [3,6,7]. The overall average strain in silicon, *ε*_avg_, can be calculated by just averaging the strain of the neutral axis over the total length of silicon. This is because the strain of the neutral axis is the average strain across the thickness due to the linear strain distribution in the thickness direction. Using *ε*_n_(*x*) to denote strain along the neutral axis of silicon as shown in Figure 1B, *ε*_avg_ is defined as:

(4) $$\varepsilon_{\text{avg}}=\frac{\int_{-L/2}^{L/2}\varepsilon_{n}(x)dx}{L}$$

Plugging Equation (3) into Equation (2) yields:

(5) $$GF={\text{GF}}_{\text{Si}}\frac{\varepsilon_{\text{avg}}}{\varepsilon_{\text{app}}}$$

suggesting that to find out the effective *GF* of the strain gauge system, we need to calculate the average strain in silicon.

The maximum strain in silicon, *ε*_max_, will be related to the stretchability of the system. We define *stretchability* as the critical strain applied to the substrate, $\varepsilon_{\text{app}}^{\text{cr}}$, beyond which silicon will rupture. For brittle silicon, we adopt a failure criterion, *ε*_max_ = *ε*_cr_, in which *ε*_max_ represents the maximum tensile strain in silicon and *ε*_cr_ denotes the intrinsic critical strain-to-rupture of silicon that is to be measured experimentally. The failure criterion can be rewritten in the normalized form $\varepsilon_{\max}/\varepsilon_{\text{app}}=\varepsilon_{\text{cr}}/\varepsilon_{\text{app}}^{\text{cr}}$, which can be rearranged to obtain the stretchability:

(6) $$\varepsilon_{\text{app}}^{\text{cr}}=\frac{\varepsilon_{\text{cr}}}{\varepsilon_{\max}/\varepsilon_{\text{app}}}$$

When the polymer substrate is very thin or very soft, there will be slight concave bending in the silicon strip when the substrate is subject to tensile strain (Figure 1C,D). As a result, the maximum strain in silicon always takes place along the bottom of silicon. Using *ε*_b_(*x*) to denote strain along the bottom of silicon as shown in Figure 1B, then:

(7) $$\varepsilon_{\max}=\max\{\varepsilon_{b}(x)\}$$

As we have related the device performance indices, *GF* and stretchability, to *ε*_avg_ and *ε*_max_ in silicon respectively, in the following we will calculate *ε*_avg_ and *ε*_max_ to ultimately determine *GF* and stretchability. Through dimensional analysis, we can get

(8) $$\frac{\varepsilon_{\text{avg}}}{\varepsilon_{\text{app}}}=f\left(\frac{{\bar{E}}_{\text{Si}}}{{\bar{E}}_{s}},\frac{L}{h},\frac{h}{H}\right)$$

and:

(9) $$\frac{\varepsilon_{\max}}{\varepsilon_{\text{app}}}=g\left(\frac{{\bar{E}}_{\text{Si}}}{{\bar{E}}_{s}},\frac{L}{h},\frac{h}{H}\right)$$

Where *Ē* = *E*/(1 − *v*^2^) represents the plane strain modulus with *v* being the Poisson's ratio. Our goal is to find out the functional forms of *f* and *g*. Several shear lag models have been built to solve similar problems of stiff thin films on compliant substrates [14,23,24], but they had to make special assumptions of shear stress distribution along the film/substrate interface. In fact, there is no uniform function of interface shear stress distribution that is applicable to wide ranges of *Ē*_Si_/*Ē*_s_, *L*/*h*, and *H*/*h*, which is the case for stretchable silicon-on-polymer strain gauges. As a result, we will first use FEM to find exact solutions for *ε*_avg_/*ε*_app_ and *ε*_max_/*ε*_app_, and then use analytical methods to derive the functional forms of Equations (8) and (9) for extreme cases (e.g., *L*/*H* ≫ 1 and *L*/*H* ≪ 1) and compare with FEM results.

## 3. Finite Element Modeling

Over one hundred finite element models are built using commercial software package ABAQUS 6.11 to reveal the effect of the three variables in Equations (8) and (9). The boundary conditions and geometries are chosen to resemble the experimental setups when the sample is subject to uniaxial tension for *GF* calibration [9,13,14]. Because of symmetry, only the right half of the system shown in Figure 1B is modeled. A symmetric boundary condition is applied along the axis of symmetry, and the right end of the substrate is subject to a uniform horizontal displacement so that *ε*_app_ is fixed to be 10% for all models. Nonlinear geometry analysis has to be enabled in ABAQUS because large deformation can be found in the portion of the polymer substrate that is not covered by silicon. Silicon thickness *h* is set to be 340 nm, the same thickness as the silicon nanomembrane used in most stretchable strain gauges [9,13,14]. Silicon length *L* varies from 10 μm to 20,000 μm and substrate thickness *H* varies from 30 μm to 3,000 μm, all are representative experimental parameters. The substrate length is set to be always 1.5 *L* for this unit cell. Perfect bonding is assumed between silicon and the substrate. Silicon and substrate are meshed using linear beam and plane-strain elements respectively. We model (110) silicon as a linear elastic material and polymer substrates as Neo-Hookean materials. Although the stress-strain curves for linear elastic and Neo-Hookean materials are not quite differentiable for strains up to 10%, we found the Neo-Hookean constitutive law is very helpful to achieve convergent solutions when nonlinear geometry analysis is enabled in ABAQUS. Effective materials properties in terms of Young's modulus and Poisson's ratio are listed in Table 1.

Representative contour plots of longitudinal strain in the substrate and in silicon are given in Figure 1C–E. They are from models with parameters *L* = 1,000 μm, *E*_s_ = 60 kPa and *H* varying from 30 μm to 3,000 μm. When the substrate is stretched by 10%, strain in silicon is as small as 10^−7^ to 10^−4^, depending on substrate thickness. It is because silicon is six orders of magnitude stiffer than Ecoflex substrate (*E*_s_ = 60 kPa) so that it is highly resistant to elongation, whereas the portion of the substrate not covered by silicon has to accommodate the applied strain by tensile strains up to 25%. Due to huge elastic mismatch between silicon and Ecoflex, horizontally applied tensile strain on a thin substrate will cause bending deformation in the silicon strip, especially near the end (Figure 1C,D). Therefore when the substrate is thin, the strain along the neutral axis of silicon, *ε*_n_, is always smaller than the strain along the bottom of silicon, *ε*_b_. Although *ε*_n_ is monotonic with *x*, *ε*_b_ might not be so due to localized bending near the end of the strip. When the substrate is thick enough (Figure 1E), it can be considered semi-infinite and hence can suppress the bending, resulting in identical *ε*_n_ and *ε*_b_. Figure 1C–E only list FEM results for *E*_s_ = 60 kPa, as the substrate becomes stiffer, the bending will be less significant.

### 3.1. Strain Distribution in Silicon

**Figure 2.**
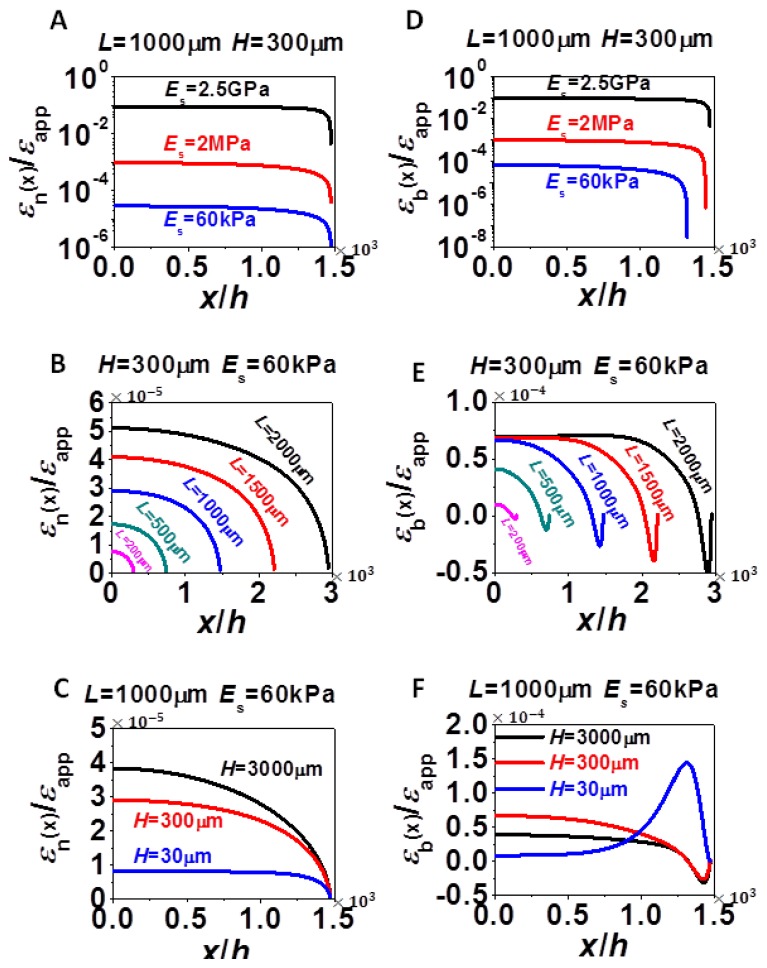
Normalized *ε*_n_ and *ε*_b_ for different *L*, *H*, and *E*_s_ combinations. (**A**) Normalized *ε*_n_(*x*) for various *E*_s_ when *L* = 1,000 μm and *H* = 300 μm are fixed. (**B**) Normalized *ε*_n_(*x*) for various *L* when *H* = 300 μm and *E*_s_ = 60 kPa are fixed. (**C**) Normalized *ε*_n_(*x*) for various *H* when *L* = 1,000 μm and *E*_s_ = 60 kPa are fixed. (**D**) Normalized *ε*_b_(*x*) for various *E*_s_ when *L* = 1,000 μm and *H* = 300 μm are fixed. (**E**) Normalized *ε*_b_(*x*) for various *L* when *H* = 300 μm and *E*_s_ = 60 kPa are fixed. (**F**) Normalized *ε*_b_(*x*) for various *H* when *L* = 1,000 μm and *E*_s_ = 60 kPa are fixed.

Figure 2 offers the distribution of *ε*_n_ and *ε*_b_ along *x* for different *E*_s_, *L*, and *H*.Figure 2A-C plot *ε*_n_ and Figure 2D-E plot *ε*_b_. In all the plots, *x* = 0 represents the center of silicon and *x* = *L*/2 represents the right edge of silicon, as defined in Figure 1B. A generic feature of every strain distribution curve in Figure 2 is that strains always vanish at the traction free edge. Because of the traction free edge, the internal normal force of silicon at position *x*, *i.e.*, *E*_Si_*hε*_n_(*x*), has to balance the integration of interfacial shear stress from position *x* to the edge of silicon, *L*/2. As shown in Figure 2A-C, *ε*_n_ gradually builds up towards the center of silicon because interfacial shear force also builds up as we move away from the edge. The rate of strain growth decreases as *x* approaches the center of silicon because the interfacial shear stress decays from the edge to the center of silicon. If *L* is large enough, a plateau of maximum tensile strain could be reached toward the center of silicon. The distribution of *ε*_b_ shares some similarity with *ε*_n_, as shown in Figure 2D-E. However, *ε*_b_ is not always monotonic with *x*. When the substrate is soft, e.g., *E*_s_ = 60 kPa, edge bending can cause large variations in *ε*_b_, as shown in Figure 2E,F.

To study one effect at a time, we first vary *E*_s_ with *L* = 1,000 μm and *H* = 300 μm fixed. The distribution of *ε*_n_ and *ε*_b_ for different *E*_s_ (60 kPa, 2 MPa, and 2.5 GPa) are plotted in Figure 2A,D, with vertical axis in logarithmic scales. A key observation is that both *ε*_n_ and *ε*_b_ increase as substrate modulus increases. It is simply because the stiffer substrate can apply higher shear stress to silicon, meaning that silicon can be stretched more by stiffer substrate. When substrate is very stiff, *i.e.*, when *E*_s_ = 2.5 GPa, there is little difference between *ε*_n_ and *ε*_b_ curves because the assembly will stay almost flat. But when *E*_s_ = 60 kPa, *ε*_b_ are larger than *ε*_n_ due to slight global concave bending.

To study the effect of *L*, we fix *H* = 300 μm and *E*_s_ = 60 kPa. The distribution of *ε*_n_ and *ε*_b_ for different *L* (200, 500, 1,000, 1,500, and 2,000 μm) are plotted in Figure 2B,E. The plateau values of both *ε*_n_ and *ε*_b_ increase as *L* increases because the longer *L* provides the longer distance for interfacial shear force (and hence the normal strain) to build up. Although *ε*_n_ monotonically decreases with increasing *x*, *ε*_b_ curves in Figure 2D always have some abnormal behaviors near the edge of silicon, as the result of highly localized curvature near the edge of silicon, as evident in Figure 1C-E.

To study the effect of *H*, we fix *L* = 1,000 μm and *E*_s_ = 60 kPa and vary *H* from 30 μm to 3,000 μm in Figure 2C,F. Since thicker substrates provide stronger constraint to silicon, the plateau value of *ε*_n_ is higher when *H* is larger. The variation of *ε*_b_ is more complicated than *ε*_n_ because when substrate is soft (e.g., *E*_s_ = 60 kPa in this case), bending strain could become very significant. Depending on substrate thickness, bending strain distribution also varies a lot. When *H* = 3,000 μm, there is very small bending curvature in the majority part of silicon (Figure 1E) and hence the plateau values of the black curves in Figure 2C,F are very similar. At the edge of silicon, localized concave bending induces compressive strain at the bottom of silicon because the neutral axis of the assembly is located somewhere within the substrate, hence a dip presents in the black curve of Figure 2F. When *H* = 300 μm, neutral axis locates within silicon, hence slight global concave bending (Figure 1D) will induce tensile strain along the bottom of silicon, resulting in elevated red curve in Figure 2F. Toward the edge of silicon, the neutral axis will gradually shift into the substrate and hence a dip also presents. When *H* = 30 μm, bending occurs almost exclusively at the edge (Figure 1C), hence the plateau values of the blue curves in Figure 2C,F are very similar. However, toward the edge of silicon, there is highly localized concave bending. Since neutral axis is within silicon, the bottom of silicon is subject to large tensile strain, resulting in a big bump in the blue curve of Figure 2F. As a conclusion, Figure 2 has demonstrated that unlike *ε*_n_(*x*), *ε*_b_(*x*) is not a monotonic function and the effect of *H* on it is not monotonic either. To find the maximum tensile strain, *ε*_max_, in silicon, we need to find the maximum positive values of each *ε*_b_(*x*) curve, which does not necessarily occur at the center of silicon.

### 3.2. Average Strain and Maximum Strain

**Figure 3.**
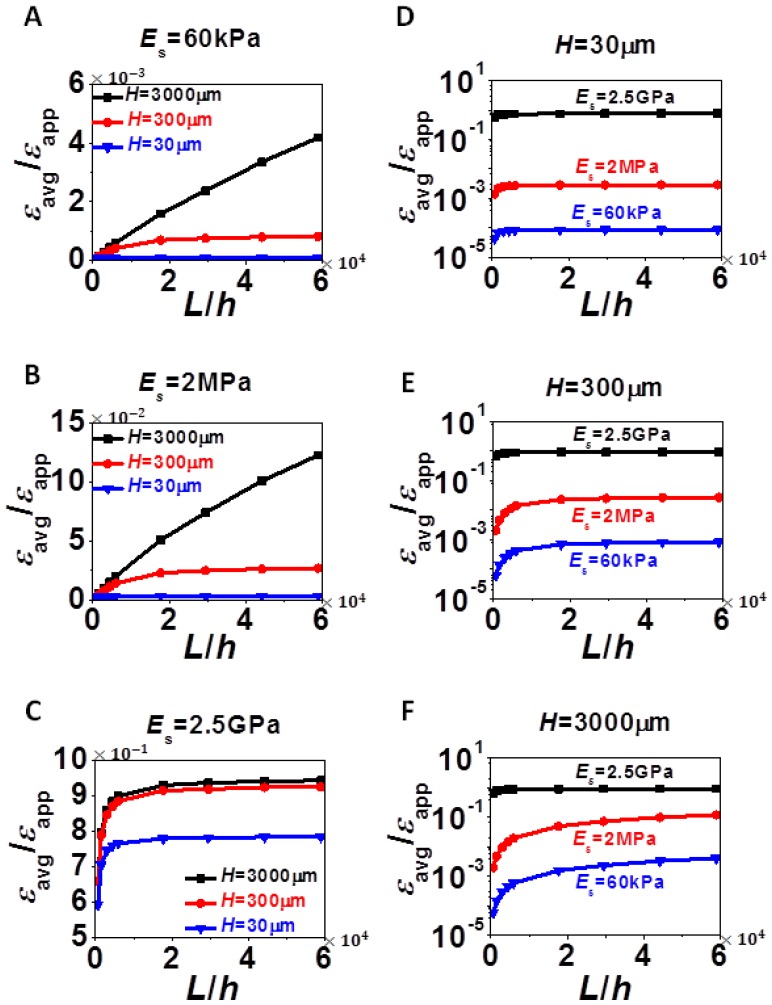
Normalized average strains in silicon as a function of normalized silicon length for various combinations of *H* and *E*_s_. (**A**) Ecoflex substrate: *E*_s_ = 60 kPa. (**B**) 10:1 PDMS substrate: *E*_s_ = 2 MPa. (**C**) Kapton substrate: *E*_s_ = 2.5 GPa. (**D**) *H* = 30 μm. (**E**) *H* = 300 μm. (**F**) *H* = 3,000 μm. Average strain in silicon increases monotonically with *L*, *H*, and *E*_s_.

**Figure 4.**
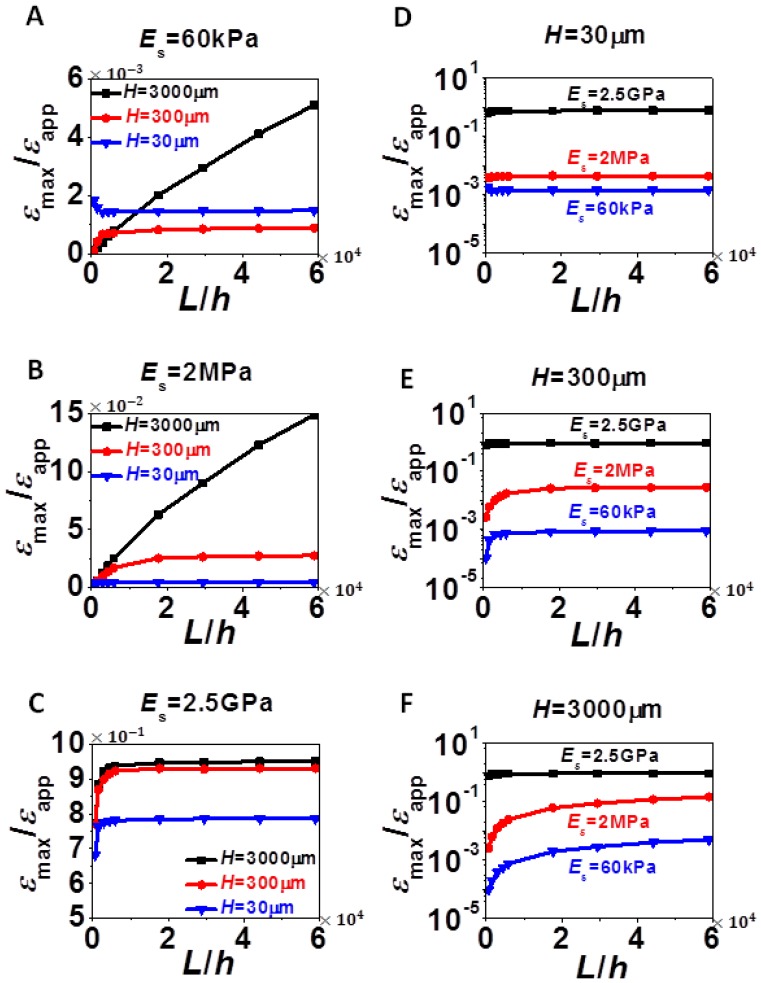
Normalized maximum strains in silicon as a function of normalized silicon length for various combinations of substrate modulus and thickness. (**A**) Ecoflex substrate: *E*_s_ = 60 kPa. (**B**) 10:1 PDMS substrate: *E*_s_ = 2 MPa. (**C**) Kapton substrate: *E*_s_ = 2.5 GPa. (**D**) *H* = *30* μm. (**E**) *H* = 300 μm. (**F**) *H* = 3,000 μm. Maximum strain in silicon increases monotonically with *L* and *E*_s_, but not with *H* when *L* and *E*_s_ are both small, as shown in (A).

With the insights from strain distribution in Figure 2, Figures 3 and 4 investigate the effect of the three variables *L*, *H*, and *E*_s_, on the average strain (*ε*_avg_) and the maximum strain (*ε*_max_) in silicon. Average strains are calculated from averaging the values of *ε*_n_ (Equation (4)) and maximum strains are determined through finding the maximum positive values of *ε*_b_ (Equation (7)). Figure 3 plots normalized *ε*_avg_ as a function of *L*/*h*. The same set of data is presented in two different ways: each plot in Figure 3A-C has a fixed *E*_s_ and varying *H* whereas each plot of Figure 3D-F has a fixed *H* and varying *E*_s_. All plots in Figure 3 show that *ε*_avg_ increases with increased film size (*L*), substrate thickness (*H*) and substrate modulus (*E_s_*), all of which imply the stronger constraint the substrate is able to apply to silicon, the higher *ε*_avg_. Due to experimental limitations on *L* and *H*, changing *E*_s_ would be the most effective way to tune *ε*_avg_, by orders of magnitude. According to Figure 3A-C, it is interesting to notice that when *L* is very small, especially compared to *H*, there exists a linear relation between *ε*_avg_ and *L*. According to Figure 3D-F, it is also easy to discover that when *L* is large enough, *ε*_avg_ will always reach a plateau, *i.e.*, *ε*_avg_ becomes independent of *L*, and the smaller the *H*, the faster the plateau can be reached. We will thereby consider the limiting cases of *L*/*H* ≪ 1 and *L*/*H* ≫ 1 and try to derive analytical solutions for *ε*_avg_ in Section 4.

Figure 4 plots the normalized *ε*_max_ as a function of *L*/*H* for various *H* and *E*_s_ in a format similar to Figure 3. Each plot in Figure 4A-C has a fixed *E*_s_ and varying *H* whereas each plot of Figure 4D-F has a fixed *H* and varying *E*_s_.

The difference between *ε*_max_ and *ε*_avg_ is that *ε*_max_ has extra bending contribution. Therefore, similar to *ε*_avg_, *ε*_max_ always increases with increased *L* and *E*_s_, and with increased H in most cases. However, when the substrate is very compliant and when *L* is small, Figure 4A shows that *ε*_max_ could be higher in thinner substrate due to localized bending effect we discussed for Figure 2F. As *E*_s_ increases to 2.5 GPa, as shown in Figure 4C, bending effect is almost negligible and hence *ε*_max_ and *ε*_avg_ become undistinguishable, *i.e.*, Figures 3C and 4C look identical. Similar conclusions can be applied to thick substrate (*H* = 3,000 μm) when comparing Figures 3F and 4F. For the two limiting cases, *L*/*H* ≪ 1 and *L*/*H* ≫ 1, we also observed similar features as in Figure 3, *i.e.*, linear when *L*/*H* ≪ 1 and plateau when *L*/*H* ≫ 1. Hence analytical solutions of *ε*_max_ will be derived in Section 4 as well.

## 4. Analytical Modeling

In this section, we are going to develop analytical models for two limiting cases: *L*/*H* ≪ 1 and *L*/*H* ≫ 1, and the analytical solutions will be compared with the FEM results.

### 4.1. When L/H ≪ 1

When *L*/*H* ≪ 1, *h*/*H* ≪ 1 is also true since experimentally *L* > *h* is always valid. In this case, *H* is no longer a relevant variable in the system and the substrate can be considered infinitely thick. Hence *ε*_avg_ and *ε*_max_ only depend on *Ē*_s_/*Ē*_Si_ and *L*/*h*, and Equation (8) degenerates to:

(10) $$\frac{\varepsilon }{\varepsilon_{\text{app}}}=f\left(\frac{{\bar{E}}_{s}}{{\bar{E}}_{\text{Si}}},\frac{L}{h}\right)$$

**Figure 5.**
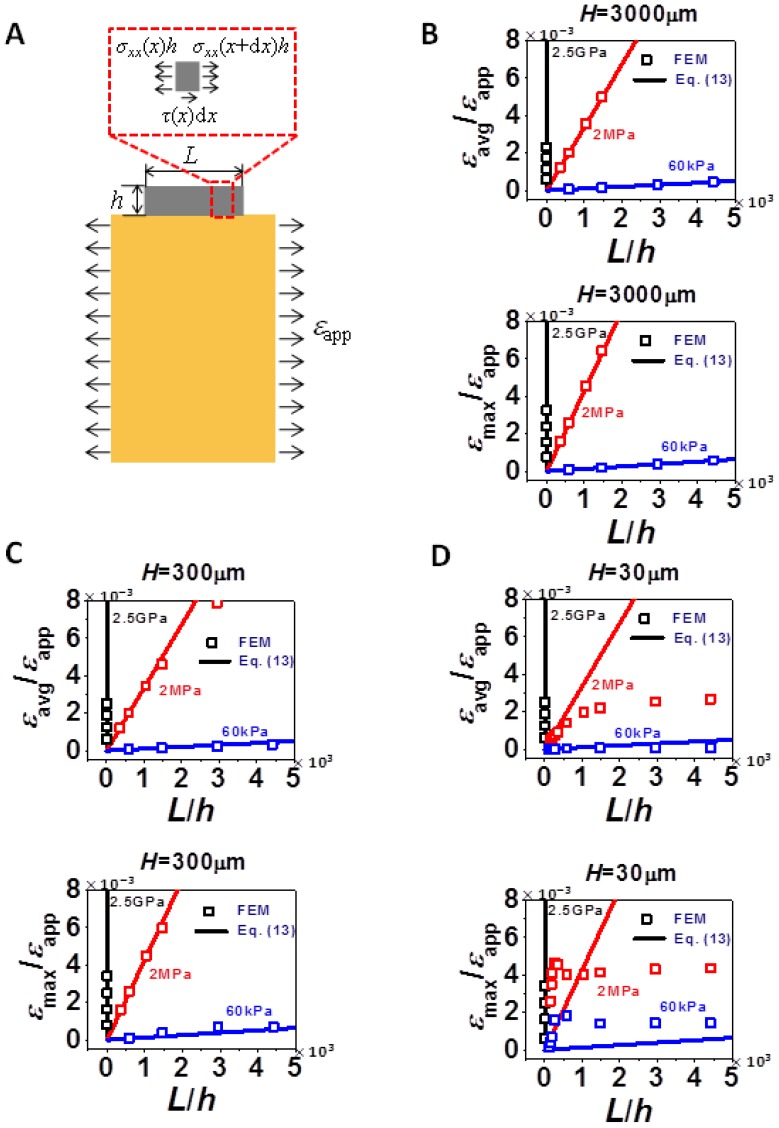
Comparison of Equation (13) and FEM results of *ε*_avg_/*ε*_app_ and *ε*_max_/*ε*_app_ when *L*/*H* ≪ 1. (**A**) Schematic of a thin silicon strip bonded to an infinitely thick substrate and the FBD of a thin slice of silicon. (**B**) *ε*_avg_/*ε*_app_ and *ε*_max_/*ε*_app_ for *H* = 3,000 μm. (**C**) *ε*_avg_/*ε*_app_ and *ε*_max_/*ε*_app_ for *H* = 300 μm. (**D**) *ε*_avg_/*ε*_app_ and *ε*_max_/*ε*_app_ for *H* = 30 μm. In general, Equation (13) works better for thicker and stiffer substrate.

To determine the *f* function, a shear lag model is adopted as shown in Figure 5A. The free body diagram (FBD) of a thin slice of silicon would give the following equilibrium equation:

(11) $$h\frac{d\sigma }{dx}=\tau (x)$$

where *τ*(*x*) represents the shear stress distribution along the silicon/substrate interface. Since *τ*(*x*) is unknown and we don't want to make an arbitrary assumption for it, it is simply acknowledged that *τ*(*x*) is proportional to the Young's modulus of the substrate, *Ē*_s_. Applying Hooke's law in silicon, *σ = Ē*_Si_*ε*, and integrating once on both sides, Equation (11) reduces to:

(12) $$\varepsilon ∼\frac{{\bar{E}}_{s}}{{\bar{E}}_{\text{Si}}}\frac{L}{h}$$

And Equation (10) becomes:

(13) $$\frac{\varepsilon }{\varepsilon_{\text{app}}}=\alpha \frac{{\bar{E}}_{s}}{{\bar{E}}_{\text{Si}}}\frac{L}{h}$$

where *α* is a proportional coefficient to be found out through fitting FEM results of small *L*'s. *α* is a generic coefficient which once fitted, should be applicable to all combinations of *L*, *H*, and *E*_s_, provided *L*/*H* ≪ 1. We choose to fit the data of strain gauges with *H* = 3,000 μm, *E*_s_ = 2.5 GPa and very small *L*. The two black curves in Figure 5B are the fitted curves and *α* is found to be 0.219 for *ε*_max_/*ε*_app_ and 0.279 for *ε*_max_/*ε*_app_. We then plot Equation (13) against FEM results of all the other combinations of *E*_s_ and *H*, as long as *L*'s are small. The results are shown in Figure 5B-D. Each figure contains the comparison of Equation (13) and FEM results for *ε*_avg_/*ε*_app_ in the upper frame and *ε*_max_/*ε*_app_ in the lower frame. It is evident that Equation (13) is able to capture very wide ranges of *E*_s_ when *H* is beyond 300 μm, for both *ε*_avg_/*ε*_app_ and *ε*_max_/*ε*_app_. When *H* = 30 μm as shown in Figure 5D, Equation (13) is able to capture *ε*_avg_/*ε*_app_ over a wide range of *E*_s_ but is only able to capture *ε*_max_/*ε*_app_ when *E*_s_ = 2.5 GPa, due to the abnormal *ε*_max_ induced by large local bending.

In conclusion, when *L*/*H* ≪ 1, average and maximum strains in silicon scale linearly with *Ē*_s_ and *L*. Since *Ē*_s_ can be easily changed by orders of magnitude as shown in Table 1, strains in silicon could also be tuned cross wide ranges.

### 4.2. When L/H ≫ 1

**Figure 6.**
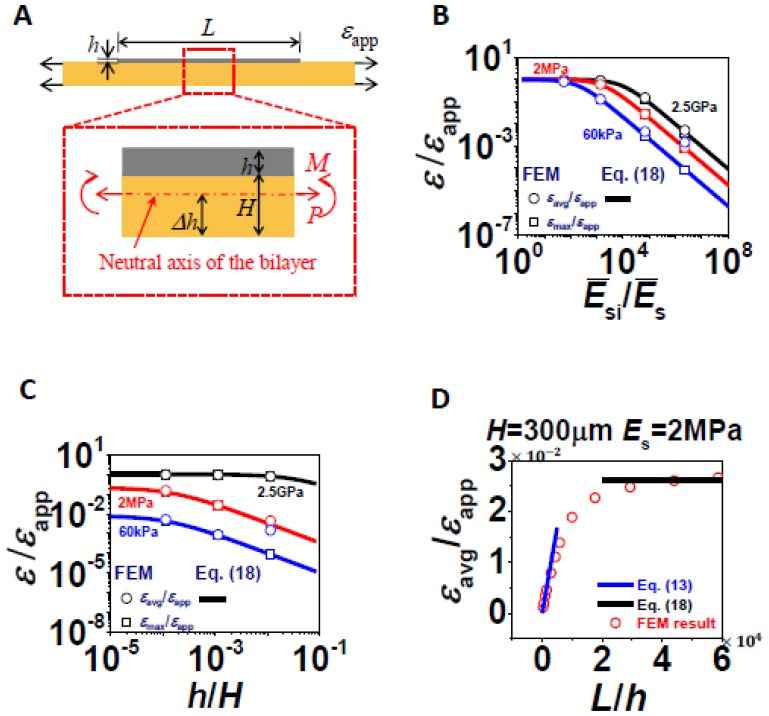
Comparison of Equation (18) and FEM results of *ε*_avg_/*ε*_app_ and *ε*_max_/*ε*_app_ when *L*/*H* ≫ 1. (**A**) Schematic of the FBD of a section of thin silicon strip bonded to polymer substrate. (**B**) *ε*_avg_/*ε*_app_ and *ε*_max_/*ε*_app_ as functions of *Ē*_Si_/*Ē*_s_. (**C**) *ε*_avg_/*ε*_app_ and *ε*_max_/*ε*_app_ as functions of *h*/*H*. (**D**) Comparison of Equations (13) and (18) against FEM for *H* = 300 μm, *E*_s_ = 2 MPa. Analytical solutions for two extreme conditions have found good agreement with FEM results.

When *L*/*H* ≫ 1, assuming *L*/*H* ≫ 1 is always valid, relevant variables reduce to *Ē*_Si_/*Ē*_s_ and *h*/*H*. To derive analytical solution for this case, we make two cross-sectional cuts within the length of silicon and the FBD is shown in Figure 6A. The neutral axis of this bilayer is given by [29]:

(14) $$\Delta =\frac{1+2\Sigma \eta +\Sigma \eta^{2}}{2\eta (1+\Sigma \eta )}$$

where *Σ* = *Ē*_Si_/*Ē*_s_, *η* = *h*/*H*.

Boundary conditions are decomposed to *P* and *M*, which are both acting at the neutral axis. Assuming the following relation between *P* and *ε*_app_:

(15) $$\varepsilon_{\text{app}}=\beta \frac{p}{{\bar{E}}_{s}H}+(1-\beta )\frac{P}{{\bar{E}}_{\text{Si}}h+{\bar{E}}_{s}H}$$

where *β* is a parameter capturing the relative contribution from uncovered substrate and (1−*β*) captures the relative contribution from the covered substrate of the silicon-substrate bilayer structure. In this paper, since substrate length is fixed to be 1.5 *L*, then ideally *β* = 1/3. But for substrate with finite thickness, *β* is assumed to have linear relationship with *H*/*h* such that:

(16) $$\beta =\frac{a}{\eta }+b$$

where *a* and *b* are constants to be fitted.

Following this practice, the simplest form of average strain in silicon can be written as:

(17) $$\varepsilon_{\text{avg}}=\frac{P}{{\bar{E}}_{\text{Si}}h+{\bar{E}}_{s}H}$$

Combining Equations (15)–(17) gives:

(18) $$\frac{\varepsilon_{\text{avg}}}{\varepsilon_{\text{app}}}=\frac{1}{1+a\Sigma +b\Sigma \eta }$$

Through curve fitting of FEM results, *a* and *b* can be obtained as *a* = 5.46 × 10^−5^ and *b* = 0.428. Equation (18) turned out to be an universal expression to capture *ε*_avg_/*ε*_app_ and *ε*_max_/*ε*_app_, over wide ranges of *Σ* and *η* provided *L* ≫ *H*. For very big *Σ* and *η*, *i.e.*, very compliant and thin substrate, Equation (18) cannot capture *ε*_max_/*ε*_app_ very well due to localized bending effects.

Figure 6B,C plots Equation (18) (as curves) against FEM results (as markers) in log log scale. Some important conclusions can be drawn and rationalized from the two plots. First, when the substrate is as stiff as silicon, or when the substrate is infinitely thick, *ε*/*ε*_app_ approaches 1. When the substrate becomes extremely soft or extremely thin, *ε*/*ε*_app_ can be reduced by orders of magnitude, and should eventually die out. Figure 6C also tells that the effect of *h*/*H* is insignificant when the substrate is very stiff but gradually becomes more effective as the substrate modulus reduces.

With the two semi-analytical solutions given by Equations (13) and (18), it is useful to plot them for a representative combination of (*Σ*, *η*) in a *L*/*H* plot as shown in Figure 6(D). It is clear that Equations (13) and (18) can successfully bond the FEM results, except the transition zone.

## 5. Tradeoff between Gauge Factor and Stretchability

**Figure 7.**
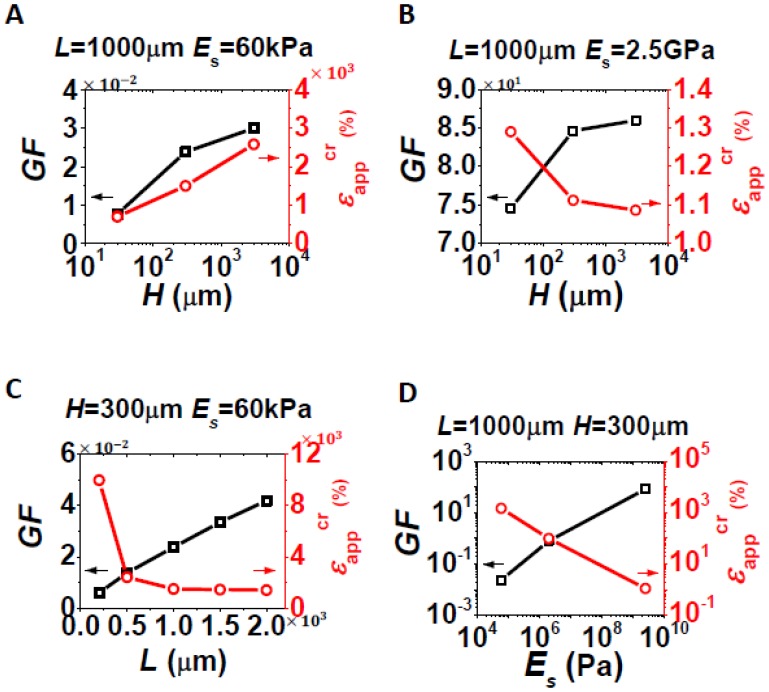
Tradeoff between *GF* and stretchability $\left(\varepsilon_{\text{app}}^{\text{cr}}\right)$: *GF* is plot in black with a vertical axis on the left and stretchability is plot in red with a vertical axis on the right. (**A**) Effect of *H* when *L* = 1 mm and *E*_s_ = 60 kPa are fixed. (**B**) Effect of *H* when *L* = 1 mm and *E*_s_ = 2.5 GPa are fixed. (**C**) Effect of *L* when *H* = 300 μm and *E*_s_ = 60 kPa are fixed. (**D**) Effect of *E*_s_ when *L* = 1 mm and *H* = 300 μm are fixed. Among the three variables, *E*_s_ has the widest range of options and hence the most significant effect.

After finding out *ε*_avg_/*ε*_app_ and *ε*_max_/*ε*_app_, the final step is to use Equations (5) and (6) to determine the gauge factor, *GF*, and the stretchability, $\varepsilon_{\text{app}}^{\text{cr}}$, of a particular strain gauge. For the purpose of illustration, we have to assume some reasonable numbers for the intrinsic properties for silicon, including *GF*_Si_ = 100 [9] and *ε*_cr_ = 1%. Some representative results are shown in Figure 7, with *GF* in black and $\varepsilon_{\text{app}}^{\text{cr}}$ in red in each plot. First, effects of *H* are studied for fixed *L*, and *E*_s_, as shown in Figure 7A,B. Comparing the two plots, *GF* always increases as *H* increases, but the variation of $\varepsilon_{\text{app}}^{\text{cr}}$ with *H* depends on *E*_s_. When the substrate is really soft and silicon is short, as the case for Figure 7A, the assembly is easy to bend especially when *H* is small. The local bending induced strain contributes significantly to *ε*_max_ which is inversely proportional to $\varepsilon_{\text{app}}^{\text{cr}}$ according to Equation (6). As a result, thinner substrate induces higher maximum strain and hence lower stretchability. When the substrate is stiff, e.g., *E*_s_ = 2.5 GPa as shown in Figure 7B, there is almost no bending in the assembly and hence the thicker substrate induces higher maximum strain (Figure 4C). It is interpreted as a tradeoff between stretchability and *GF*. As constraints from the substrate become stronger, *i.e.*, as *L*, *H*, or *E*_s_ increases, the larger strain in silicon suggests the higher *GF* but the lower stretchability, which has been confirmed by Figure 7C,D. Effects of *L* are studied for fixed *H* and *E*_s_, as shown in Figure 7C. For longer strip, the *GF* is larger but the stretchability is lower. This is always the case for other *H* and *E*_s_ combinations. Similar effects are observed for *E*_s_, as shown in Figure 7D, except that the scales of both *GF* and $\varepsilon_{\text{app}}^{\text{cr}}$ are in log scale, suggesting a big tuning range. For example, when *E*_s_ = 60 kPa, the critical applied strain to rupture silicon $(\varepsilon_{\text{app}}^{\text{cr}})$ can be as high as 10,000% in theory, which implies that the stretchability of the strain gauge is only limited by the stretchability of the substrate. But it corresponds to a *GF* as low as 0.006. Practically, *GF* should be at least 1 for strain measurement, which implies *E*_s_ should be 2 MPa and beyond, which still corresponds to stretchability beyond 100%.

The goal of plots in Figure 7 is to provide guidance for engineers to choose the right strain gauge systems under certain constraints or to predict the performance of a given stretchable strain gauge based on silicon nanomembrane. For example, to measure an object which deforms up to 30%, we will need to choose experimentally appropriate *L*, *H*, and *E*_s_ to make a strain gauge with a stretchability of 30% and maximized *GF*. Since the effects of *L* and *H* are within one order of magnitude as shown in Figure 7A–C, we could fix them at reasonable numbers and the key is to select the right stiffness for the substrate, using a plot as shown in Figure 7D. A stretchability of 30% corresponds to a *E*_s_ = 1.1 × 10^7^ Pa, which in turn predicts the *GF* to be 3.27.

We have three final remarks on applying the analysis in this paper to real strain gauge samples [9,13,14]. In real samples, brittle silicon strip is usually sandwiched between two layers of insulating polyimide, and the strip is connected to metallic interconnects (usually in serpentine shape) for data acquisition. Laminating insulating polymers on silicon strip is equivalent to an increase of *h*, which would decrease strain in silicon and hence decrease the system *GF*. Adding interconnects to silicon is equivalent to an increase of *L*, which would increase strain in silicon and hence increase the system *GF*. Therefore the idealized model in Figure 1B may not offer the most accurate quantitative prediction for a practical sample. But we can always run 3D FEM to calculate the strain in silicon taking into account the actual layout of the circuits and the multilayer lamination to predict a pertinent *GF* and stretchability for that specific strain gauge system.

The coupling between tension and bending is a generic problem associated with mechanical strain gauges. This study has also shed light on this issue. When *E*_s_ ≪ *E*_Si_, the neutral axis of the bilayer almost overlaps with the neutral axis of silicon. In this case bending will induce minimum *ε*_avg_ in silicon and hence bending effect can be neglected. When *E*_s_ is close to *E*_Si_, the neutral axis of the bilayer lies far away from the neutral axis of silicon. In this case the bending will induce large *ε*_avg_ in silicon. To minimize the bending induced signal when the substrate is a stiff polymer, one can add an identical polymer layer on top of silicon, forming a sandwiched structure to locate silicon along the neutral axis of the sandwich structure. This structure can be readily analyzed by replacing the old *H* with the new 2*H*, leaving everything else unchanged.

Due to the mismatch in coefficients of thermal expansion (CTE) between silicon and polymer, temperature variation will induce stress and hence resistance change in silicon. The best known method to eliminate temperature effect is to use Wheatstone bridge instead of single resistors. The mechanical analysis on a single resistor offered in this paper is readily applicable to each linear arm of the Wheatstone bridge.

## 6. Conclusions

In conclusion, we performed strain analysis on polymer-bonded thin silicon strips using both FEM and analytical methods. The gauge factor and stretchability of a silicon-on-polymer strain gauge have been predicted as functions of the normalized length of silicon, and the normalized thickness and modulus of the polymer substrate. In general, we found that the longer strip, the thicker or the stiffer substrate will transfer a larger fraction of the applied strain to silicon. Silicon length and substrate thickness has only moderate effects on strain in silicon whereas varying the stiffness of the substrate could change the strain and hence the gauge factor and stretchability by orders of magnitude. A tradeoff between *GF* and stretchability has been discovered. Since wide ranges of gauge factor and stretchability can be achieved through tuning the substrate modulus, results from this work can be used as guidelines to design appropriate strain gauges or to predict the performance of fabricated strain gauges.

## Figures and Tables

**Table 1.** Elastic properties of materials used in FEM.

**Part**	**Element Type**	**Material**	**Young's Modulus (*E*)**	**Poisson's Ratio (*v*)**
silicon	beam	(110) Silicon [25]	169 GPa	0.27
		Ecoflex [26]	60 kPa	0.49
substrate	plane strain	10:1 PDMS [27]	2 MPa	0.49
		Polyimide [28]	2.5 GPa	0.34

## References

[1] Window A.L. (1992). Strain Gauge Technology.

[2] Lu N.S., Wang X., Suo Z.G., Vlassak J. (2007). Metal films on polymer substrates stretched beyond 50%. Appl. Phys. Lett..

[3] Smith C.S. (1954). Piezoresistance effect in germanium and silicon. Phys. Rev..

[4] Sze S.M. (1994). Semiconductor Sensors.

[5] Barlian A.A., Park W.T., Mallon J.R., Rastegar A.J., Pruitt B.L. (2009). Review: Semiconductor piezoresistance for microsystems. IEEE Proc..

[6] Kanda Y. (1991). Piezoresistance effect of silicon. Sens. Actuators A Phys..

[7] Middelhoek S. (1994). Silicon Sensors.

[8] Apanius C., Estrada H.V., Nagy M.L., Siekkinen J.W. A User-Friendly, High-Sensitivity Strain Gauge. http://www.sensorsmag.com/sensors/force-strain-load-torque/a-user-friendly-high-sensitivity-strain-gauge-1091.

[9] Won S.M., Kim H.S., Lu N.S., Kim D.G., del Solar C., Duenas T., Ameen A., Rogers J.A. (2011). Piezoresistive strain sensors and multiplexed arrays using assemblies of single-crystalline silicon nanoribbons on plastic substrates. IEEE Trans. Electron. Devices.

[10] Leonardi M., Pitchon E.M., Bertsch A., Renaud P., Mermoud A. (2009). Wireless contact lens sensor for intraocular pressure monitoring: Assessment on enucleated pig eyes. Acta Ophthalmol..

[11] Lu N., Lu C., Yang S., Rogers J. (2012). Highly sensitive skin-mountable strain gauges based entirely on elastomers. Adv. Funct. Mater..

[12] Yamada T., Hayamizu Y., Yamamoto Y., Yomogida Y., Izadi-Najafabadi A., Futaba D.N., Hata K. (2011). A stretchable carbon nanotube strain sensor for human-motion detection. Nat. Nanotechnol..

[13] Ying M., Bonifas A.P., Lu N.S., Su Y.W., Li R., Cheng H.Y., Ameen A., Huang Y.G., Rogers J.A. (2012). Silicon nanomembranes for fingertip electronics. Nanotechnology.

[14] Kim D., Ghaffari R., Lu N., Wang S., Lee S.P., Keum H., D'Angelo R., Klinker L., Su Y., Lu C. (2012). Electronic sensor and actuator webs for large-area complex geometry cardiac mapping and therapy. Proc. Natl. Acad. Sci. USA.

[15] Liu C. (2007). Recent developments in polymer MEMS. Adv. Mater..

[16] Dharap P., Li Z.L., Nagarajaiah S., Barrera E.V. (2004). Nanotube film based on single-wall carbon nanotubes for strain sensing. Nanotechnology.

[17] Sakhaee-Pour A., Ahmadian M.T., Vafai A. (2008). Potential application of single-layered graphene sheet as strain sensor. Solid State Commun..

[18] Rogers J.A., Lagally M.G., Nuzzo R.G. (2011). Synthesis, assembly and applications of semiconductor nanomembranes. Nature.

[19] Lu N.S., Yoon J.I., Suo Z.G. (2007). Delamination of stiff islands patterned on stretchable substrates. Int. J. Mater. Res..

[20] Yoon J., Zhang Z., Lu N.S., Suo Z.G. (2007). The effect of coating in increasing the critical size of islands on a compliant substrate. Appl. Phys. Lett..

[21] Sun J.Y., Lu N.S., Yoon J., Oh K.H., Suo Z.G., Vlassak J.J. (2009). Inorganic islands on a highly stretchable polyimide substrate. J. Mater. Res..

[22] Liu C. (2006). Foundations of MEMS.

[23] Xia Z.C., Hutchinson J.W. (2000). Crack patterns in thin films. J. Mech. Phys. Solids.

[24] Handge U.A. (2002). Analysis of a shear-lag model with nonlinear elastic stress transfer for sequential cracking of polymer coatings. J. Mater. Sci..

[25] Hopcroft M.A., Nix W.D., Kenny T.W. (2010). What is the young's modulus of silicon?. J. Microelectromechanical Syst..

[26] Kim D.H., Lu N.S., Ma R., Kim Y.S., Kim R.H., Wang S.D., Wu J., Won S.M., Tao H., Islam A. (2011). Epidermal electronics. Science.

[27] Fuard D., Tzvetkova-Chevolleau T., Decossas S., Tracqui P., Schiavone P. (2008). Optimization of poly-di-methyl-siloxane (PDMS) substrates for studying cellular adhesion and motility. Microelectron. Eng..

[28] DuPont™ Kapton® HN Polyimide Film Technical Data Sheet. http://www2.dupont.com/Kapton/en_US/assets/downloads/pdf/HN_datasheet.pdf.

[29] Hutchinson J.W., Suo Z. (1992). Mixed-mode cracking in layered materials. Adv. Appl. Mech..

